# Drug Induced Sleep Endoscopy in Obstructive Sleep Apnea

**Published:** 2018-02

**Authors:** Mohammad Reza Sharifian, Mohammad Zarrinkamar, Mohammad Sadegh Alimardani, Mehdi Bakhshaee, Hadi Asadpour, Negar Morovatdar, Mahnaz Amini

**Affiliations:** 1 Sinus and Surgical Endoscopic Research Center, Faculty of Medicine, Mashhad University of Medical Science, Mashhad, Iran; 2 Department of Othorhinolaryngology-Head and Neck Surgery, Faculty of Medicine, Mashhad University of Medical Science, Mashhad, Iran; 3 Department of Psychiatry, School of Medicine, Sleep Clinic of Ebn-e-Sina Hospital, Psychiatry and Behavioral Sciences Research Center, Mashhad University of Medical Sciences, Mashhad, Iran; 4 Department of Community Medicine, Clinical Research Unit, Mashhad University of Medical Sciences, Mashhad, Iran; 5 Department of Pulmonary and Critical Care Medicine, Lung Diseases Research Center, Faculty of Medicine, Mashhad University of Medical Science, Mashhad, Iran.

**Keywords:** Obstructive sleep apnea, Drug-induced sleep endoscopy, Upper airway collapse, Apnea hypopnea index

## Abstract

**Background:**

One of the main challenges of surgical treatment in Obstructive Sleep Apnea (OSA) is identifying the correct site of upper airway obstruction in an individual patient. Drug-Induced Sleep Endoscopy (DISE) in sedated patients with obstructive sleep apnea is the technique of choice for revealing anatomic and dynamic collapsible areas.

**Materials and Methods:**

In a prospective cross-sectional study adult patients with OSA documented by polysomnography were evaluated by sleep endoscopy. DISE had been done by an otolaryngologist in the setting of operating room during infusion of propofol and after the start of snoring. Endoscopic findings were recorded and evaluated from the aspect of obstruction level, severity, and multiplicity.

**Results:**

Twenty OSA patients (60% men) with mean±SD age of 38.9±9.26 years and mean Body Mass Index (BMI) of 26.57 kg/m2 were included in the study. OSA was severe in 11(55%) and moderate in 5(25%) subjects. Unilevel airway collapse was observed as retropalatal in 4(20%) and retrolingual in 3(15%) subjects. Multilevel collapse had been observed in the other 13(65%) patients. Most patients (65%) had multilevel obstruction especially those with BMI>30 (p<0.05). With increasing BMI, obstruction changed from unilevel to multilevel. None of the subjects showed complications with propofol or endoscopy procedure.

**Conclusion:**

Our study showed DISE is safe, easy to perform, and informative in OSA patients. In particular, we observed a significant association between obesity and multilevel upper airway collapse.

## INTRODUCTION

Drug-Induced Sleep Endoscopy (DISE) as a validated study is widely used in determining surgical approach in Obstructive Sleep Apnea (OSA) (1). Although endoscopy during physiologic sleep is the ideal procedure for evaluating the site of airway collapse, but it is unpleasant to patient and impractical for physician. Drug-induced sleep endoscopy has been introduced in 1991 in order to overcome these limitations (2).

Physiology of airway collapse in wake state has been shown to be different from induced sleep with only 25% coherence between results of wake and sleep endoscopy (1). Mapping of dynamic upper airway collapse during sleep is a key point in OSA patients who are candidate for surgical treatment (3). Although induced sleep has its own drawbacks DISE has been validated as a useful tool in improving surgical success (4,5).

We planned this study to assess safety of DISE in our population and to compare the results of clinical and diagnostic evaluations with those of sleep endoscopy. We also evaluated the correlation between clinical parameters and levels and patterns of obstruction..

## MATERIALS AND METHODS

### Study design and participants

This is a prospective cross-sectional study on patients diagnosed as OSA admitted to sleep clinic of Ebn-e-Sina University Hospital from January to July 2016, Mashhad, Iran. OSA had been diagnosed by either full-night or split-night Polysomnography (PSG). PSG results were scored by an experienced sleep medicine specialist according to AASM 2015 criteria with OSA defined as Apnea-Hypopnea Index (AHI) more than 5 per hour of sleep plus daytime symptoms or AHI>15. OSA severity was classified as mild (AHI=5–14), moderate (AHI=15–29) and severe (AHI≥30).

Subjects with the following criteria were excluded from the study: severe bilateral nasal obstruction preventing pass of the endoscope, known history of propofol allergy, history of upper respiratory tract infection in past 2 weeks and unwillingness to participate in the study.

### Baseline clinical assessments

After clarifying the aims and methods of the study and subscribing a written informed consent by the patients, they received a full otolaryngologic examination to evaluate wake status of upper airway and in particular size of uvula, soft palate and tonsils, tongue base, vallecula; size and shape of epiglottis, the degree of upper airway crowding (scored as Mallampati score) and state of mandibular occlusion (retrognathia, prognathia or normal occlusion). Although subjects were not necessarily candidates for surgical treatment of OSA, they were examined by an anesthesiologist before preparing for DISE. Patients were asked to sleep at least 6 hours the night before scheduled DISE day.

Demographic characteristics [including age, sex and Body Mass Index (BMI)] along with data on the level of daytime sleepiness assessed by Epworth Sleepiness Scale (ESS) were gathered. Patients were categorized as sleepy and non-sleepy according to their ESS score (0–9 and 10–24, respectively).

### Drug Induced Sleep Endoscopy Procedure

DISE was done in the setting of an operating room while airway access and intubation materials were ready. Patient lied down supine on the operating table with their neck in the neutral position. After reassuring the patient about short length of the procedure and checking their vital signs, propofol infusion had been started by an infusion pump (loading dose 2 mg/kg and maintenance dose 50μg/kg/hr) via an antecubital vein. Maintenance dose of propofol had been continued till snoring occurred. The anesthesiologist tightly monitored patient’s breaths and palpated upper abdomen to find if there is any cessation of diaphragmatic motion as the sign of temporary central sleep apnea at sleep start. After resolution of central apnea and start of snoring a flexible nasopharyngoscope (Olympus, Japan) was passed through one of the nostrils, whichever more patent, and pushed down to larynx. The otolaryngologist examined the whole pass for finding the level of soft tissue vibration (as the site of snoring sound production) and/or airway collapse (as the site of obstructive apnea).

In the case of severe desaturation (O2 saturation<88%) mandibular advancement maneuver (jaw thrust) had been done by anesthesiologist to resolve the obstructive event temporarily.

### Outcome measures

Our main outcome measure was the level of upper airway obstruction. Level of obstruction was defined as retropharyngeal, retrolingual, retropalatal and type of obstruction as circular, antero-posterior and latero-lateral. We also classified the subjects according to the number of obstruction levels as unilevel and multilevel (more than one level of collapse) and complete and incomplete (partial) collapse.

### Statistical analysis

Statistical analysis was performed with SPSS (version 16, SPSS Inc., IL, and USA). Normality of data was assessed by Kolmogorov Smirnov test. Continuous variables according to normal distribution were tested via independent sample t test. Chi Square test was used for categorical variables. Kappa agreement was used for assessing the level of agreement between two categorical variables. Two sided p-value <0.05 was considered to be statistically significant.

## RESULTS

### Demographic and anthropometric results:

Thirty-two subjects with OSA were included in the study. Twelve subjects (not subscribing written consent) were excluded. Of the remaining 20 patients with age range of 26 to 63 years (mean 38.9±9.26) and mean BMI 26.57kg/m2, 12 (60%) were male (Table 1).

**Table 1.** Baseline characteristics of patients according to the level of obstruction

**Variables**	**Obstruction level**		***p*-value**

	**Unilevel**	**Multilevel**	
**Age (yr)**	35.29±8.03	40.85±9.59	0.29
**Male gender**	5 (71.4%)	7 (53.8%)	0.44
**BMI<30**	7 (100%)	4 (30.8%)	0.003^*^
**ESS**≥**10**	3 (43%)	10 (76%)	0.17
**Complete collapse**	10 (76.9%)	7 (42.8 %)	0.45

* p-value <0.05 had been considered as significant level for Mann Whitney and Chi square tests; BMI: Body Mass Index; ESS: Epworth Sleepiness Scale

Most of the subjects were in the sleepy (65%) and severe OSA groups (55% severe, 25% moderate and 20% mild OSA). There was no significant difference in age and sex distribution of patients in different OSA severity classes. There was a significant association between BMI and OSA severity. In severe OSA group, higher BMI was more frequent (72.7 vs. 20% in moderate and 0% in mild OSA groups; p=0.003). No one had mandibular malocclusion.

### Level and type of obstruction:

**Figure 1.**
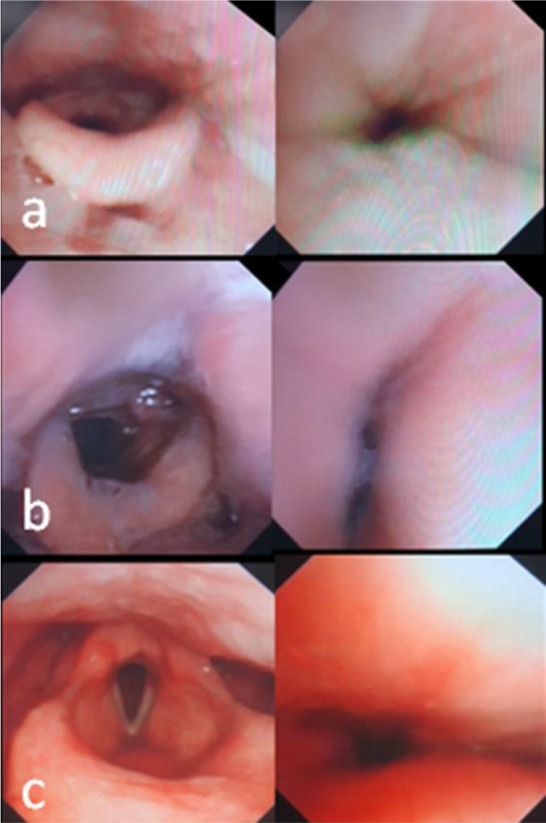
Endoscopic view of some patients undergoing DISE showing different types of upper airway collapse before (left) and after obstruction (right panel): a) circular, b) latero-lateral and c) antero-posterior collapse; DISE: Drug Induced Sleep Endoscopy.

**Figure 2.**
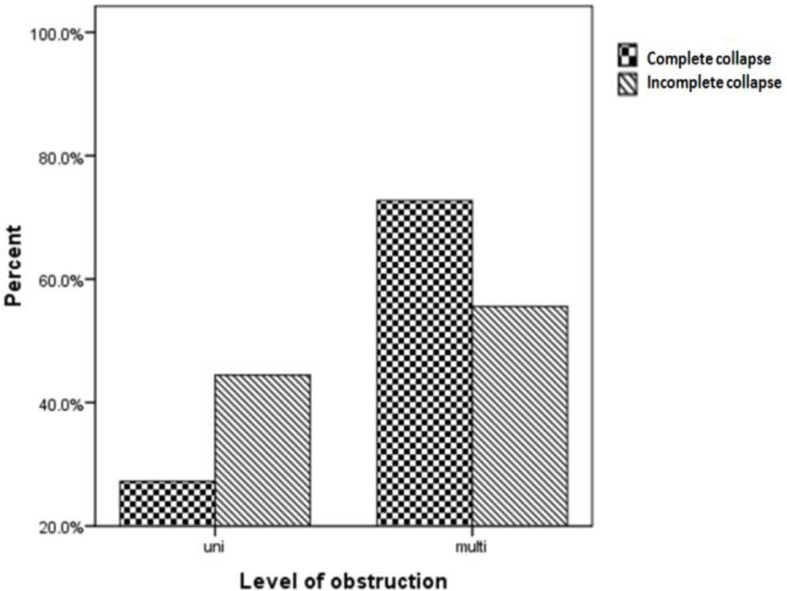
Severity of obstruction according to the level of obstruction (unilevel vs. multilevel).

Multilevel collapse was observed in 13 subjects (65%). The site of obstruction in those with unilevel collapse was retropalatal and retrolingual (20% and 15% of total subjects, respectively). The most common (50%) place of obstruction in cases with BMI<30 was retrolingual, while 100% of cases with BMI>30 showed multilevel obstruction (p=0.02). With increase in BMI the place of obstruction moved from unilevel retropalatal to multilevel obstruction. Type of obstruction was as follows: 14(70%), 4(20%) and 2(10%) showed circular, antero-posterior and latero-lateral collapse. Figure 1 shows endoscopic views of different collapse types. Most of the subjects with multilevel obstruction had complete airway collapse (Figure 2). None of the subjects showed complications with propofol or endoscopy procedure.

### Sleepiness results

Seventy-two percent of subjects with severe OSA were placed in sleepy group (ESS≥10), but OSA severity was not different between sleepy and non-sleepy groups (p=0.45). There was no significant correlation between the level of obstruction and ESS. We also found weak agreement between ESS and AHI (kappa=0.18; p=0.45).

## DISCUSSION

To the best of our knowledge this is the first report of DISE in OSA subjects in an Iranian population.

Our study reproduced the results of previous studies in terms of obstruction level (6). The most frequently involved level in our cases was retropalatal followed by retrolingual with a minority of subjects showing partial (incomplete) collapse. Salamanca et al. (7) recently published a large series of patients without including partial sites of obstruction. Bachar et al. (8) recently proposed a novel grading system for quantifying upper airway obstruction including partial sites. Circular collapse was the most common type of airway collapse in our cases. Other studies reported antero-posterior (9) and latero-lateral (10) as the most common type.

In our study obesity was significantly associated with multilevel obstruction. Abdullah et al. also observed a trend of higher BMI with≥4 sites of obstruction compared to single-site obstruction (6).

Despite introduction of Continuous Positive Airway Pressure (CPAP) as the gold standard of OSA treatment for many years, a significant number of patients (30 to 50%) do not tolerate CPAP (4,11) and some of them seek for surgical therapy as an alternative solution. The surgeon should consider DISE results before planning the optimal treatment. DISE has been recently shown (12) to have acceptable test-retest reliability which improved success rates of surgical OSA treatments.

This study showed the appropriateness and reliability of the results obtained by DISE with their correlates obtained by polysomnography in an Iranian population. Follow up study focusing on correlation of DISE results with surgical outcomes will determine its clinical importance more.

## CONCLUSION

Our data suggest that DISE is safe and easy to perform, as previously reported. Furthermore, we found a good correlation between DISE findings and clinical characteristics such as BMI in agreement with literature data.
